# Subdominant Gag-specific anti-HIV efficacy in an HLA-B^∗^57-positive elite controller

**DOI:** 10.1097/QAD.0000000000001022

**Published:** 2016-03-07

**Authors:** Ellen M. Leitman, Christian B. Willberg, Andrew De Burgh-Thomas, Hendrik Streeck, Philip J.R. Goulder, Philippa C. Matthews

**Affiliations:** a Department of Paediatrics; b Nuffield Department of Medicine, University of Oxford, Oxford; c Gloucestershire Royal Hospital, Gloucester, UK; d Institute for HIV Research, University Hospital Essen, University Duisburg-Essen, Essen, Germany; e HIV Pathogenesis Programme, The Doris Duke Medical Research Institute, University of KwaZulu-Natal, Durban, South Africa; f Department of Infectious Diseases and Microbiology, Oxford University Hospitals NGS Trust, John Radcliffe Hospital, Oxford, UK.

Despite the discovery of HIV over three decades ago, the 2008 ‘Berlin patient’ is the only case of sustained HIV remission. Other cases of apparent ‘cure’ eventually relapsed [1] and although early antiretroviral therapy (ART) has recently gained traction as a factor contributing to remission [2,3], most cases are likely to relapse [4]. In contrast, relapse in ‘elite controllers’ of HIV infection is less common. These are ART-naïve individuals who spontaneously suppress viremia to undetectable levels. Approximately 40% of elite controllers express HLA-B^∗^57 [5], an example being the original 1999 ‘Berlin patient’, in whom virologic control has been maintained for >15 years to date [6].

Mechanisms proposed to explain HLA-B^∗^57-mediated immune control include immunodominant CD8^+^ T-cell-targeting of multiple conserved Gag epitopes from which mutational escape is detrimental to viral fitness, characteristics of the T-cell receptor on HLA-B^∗^57-restricted CD8^+^ T cells, HLA-B^∗^57-peptide binding affinity, and HLA-B^∗^57 cross-talk with innate immune cells [7]. However, some HLA-B^∗^57-positive elite controllers have no detectable Gag-specific responses without ex-vivo expansion [8,9]. Here, we studied one such elite controller, to determine whether the immunodominant CD8^+^ T-cell response in such cases mediates the most potent antiviral efficacy, as Gag-specific CD8^+^ T-cell responses typically have greater capacity to inhibit viral replication than non-Gag specificities [10,11].

An African-Caribbean female was recruited in the UK at 52 years of age in 2013. She had been diagnosed with HIV in 1991, an estimated 2 years after heterosexual transmission in Jamaica (and hence is referred to here as the ‘1991 Jamaica patient’). Our study was approved by the Oxford Research Ethics Committee and the patient provided written informed consent.

For more than 24 years, she has remained ART-naïve and aviremic with a healthy CD4^+^ T-cell count (median 1237 cells/μl) (Fig. 1a). Despite being HLA-B^∗^57 : 03-positive, she demonstrated only two HIV-specific CD8^+^ T-cell responses detectable by ELISPOT assay, neither greater than 60 spot forming units (SFC)/million peripheral blood mononuclear cell (PBMC) and none detectable by tetramer staining (Fig. 1b and c). This is in contrast to the ‘1999 Berlin patient’, who had a dominant HLA-B^∗^57-restricted Nef-HW9 response of 3000 SFC/million PBMC [6] (Fig. 1b). One of the two significant ELISPOT responses in the 1991 Jamaica patient was also against this same Nef-HW9 epitope (Fig. 1b). However, via peptide stimulation of memory T-cell responses [8], we identified five HLA-B^∗^57-restricted responses (Fig. 1c), three of which we tested for their ability to inhibit HIV replication. Bulk CD8^+^ T cells demonstrated weak ex-vivo ability to suppress viral replication (Fig. 1d and e), fitting the profile of a subset of HLA-B^∗^57-positive elite controllers [12]. Of the three expanded HLA-B^∗^57-restricted CD8^+^ T-cell specificities tested, Gag-TW10-specific CD8^+^ T cells were significantly the most potent in suppressing HIV replication, followed by Nef-KF9 and then Nef-HW9 (Fig. 1d and e).

**Fig. 1:**
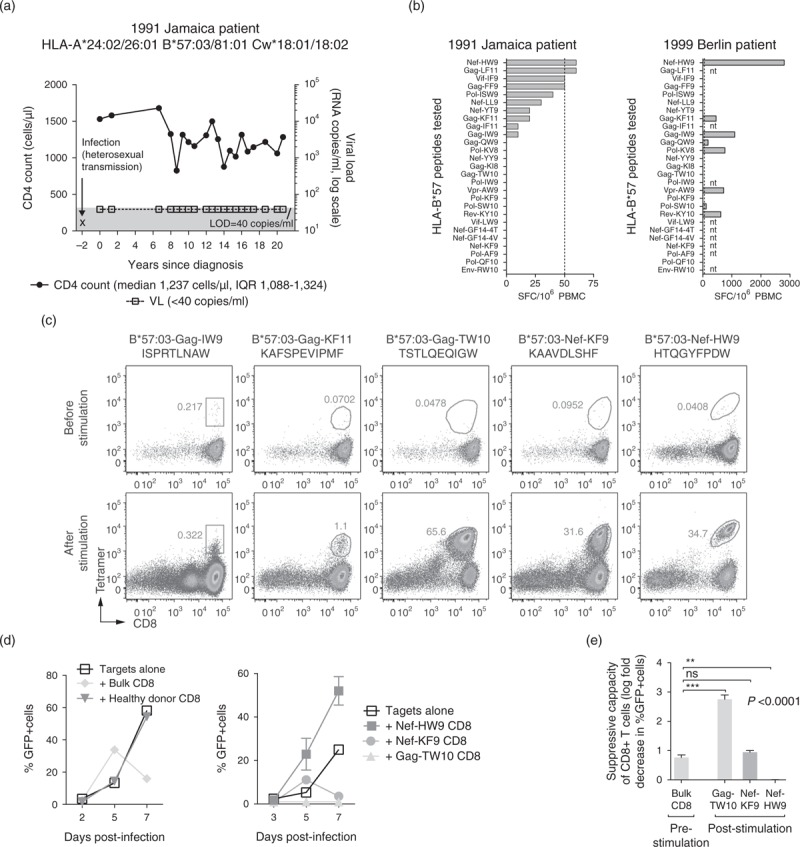
Clinical profile and anti-HIV suppressive activity of the 1991 Jamaica patient.

(a) CD4^+^ T-cell count and HIV RNA viral load measurements. ‘0’ is time of diagnosis. Limit of detection (LOD) for viral load is 40 copies/ml (gray area) and measurements are shown at 40 copies/ml for convenience. Although sequencing was unsuccessful due to lack of circulating virus, the 1991 Jamaica patient was likely infected with subtype-B HIV predominant in Jamaica. (b) ELISPOT CD8^+^ T-cell responses in unstimulated peripheral blood mononuclear cells (PBMCs) in the 1991 Jamaica patient (22 years postdiagnosis) to subtype-B consensus HLA-B^∗^57-restricted defined optimal epitopes. Responses were considered positive if they were at least three times the mean number of spot forming colonies (SFC) in the four negative control wells and had to be greater than 50 SFC/10^6^ PBMC (dotted line). CD8^+^ T-cell responses for the 1999 Berlin patient are shown to highlight the different patterns of responses (not for direct comparisons as the assays were done in different laboratories at different times). Nt = not tested. (c) PBMC (23 years postdiagnosis) were stimulated with a panel of 30 HLA-B^∗^57/81 : 01-restricted optimal peptides. Five previously undetectable HLA-B^∗^57-restricted responses were discovered poststimulation, but no HLA-B^∗^81 : 01-restricted responses. Three of these five responses were successfully expanded and tested in (d). Gated on live CD3^+^CD4^–^ cells around CD8^+^tetramer+ population; numbers indicate % tetramer+CD8^+^ cells (of CD3^+^CD4^–^). (d) Viral replication in HLA-B^∗^57 : 03-expressing H9 cells infected with NL4-3-GFP and cultured alone (‘targets alone’), with unstimulated bulk CD8^+^ T cells (left) or stimulated epitope-specific CD8^+^ T cells (right) of the 1991 Jamaica patient (23 years postdiagnosis). Each symbol represents the mean of three replicates, error bars represent the SEM. (e) Suppressive capacity (log_10_ fold decrease in % of infected GFP^+^ cells) of bulk unstimulated CD8^+^ T cells or stimulated epitope-specific CD8^+^ T cells of the 1991 Jamaica patient. Results were compared with ‘bulk CD8’ (ANOVA with Dunnett's multiple comparison post-test). ^∗^ *P* < 0.05, ^∗∗^ *P* < 0.01, ^∗∗∗^ *P* < 0.001, ns = not significant (*P* > 0.05).

The study of this HLA-B^∗^57-positive individual confirms that, in spite of HIV-specific responses being low frequency or undetectable by tetramer staining or ELISPOT assay, strong responses could be ‘recalled’ from memory, as previously reported [8]. Among these, Gag-TW10-specific CD8^+^ T cells were more potent at inhibiting viral replication than Nef-KF9-specific cells, despite the latter being a stronger response in ELISPOT assays. These data support previous findings in subjects chronically infected with HIV [13] indicating that subdominant responses may be more efficacious in terms of control of viremia. The findings here are extended also to the case of an HLA-B^∗^57-positive elite controller. Although, like the 1999 Berlin patient, this is a single case report, the data are consistent with the hypothesis that HLA-B^∗^57-mediated Gag-specific targeting by CD8^+^ T cells confers benefit to the host in HIV infection [7,14] and that vaccine induction of broad Gag-specific CD8^+^ T-cell responses would tend to increase immune control in HIV infection [15].

## Acknowledgements

The authors would like to thank the 1991 Jamaica patient for her participation in our study. The following reagent was obtained through the NIH AIDS Reagent Program, Division of AIDS, NIAID, NIH: CD3.4 Bi-specific Monoclonal Antibody (Cat#12278) from Drs Johnson Wong and Galit Alter.

Funding: This work was supported by NIHR and OUCAGS to PCM; by the National Institutes of Health [R01AI46995 to PJRG]; and by the Clarendon Foundation to EML.

### Conflicts of interest

There are no conflicts of interest.

## References

[1] Henrich TJ Hanhauser E Marty FM Sirignano MN Keating S Lee TH **Antiretroviral-free HIV-1 remission and viral rebound after allogeneic stem cell transplantation: report of 2 cases** . *Ann Intern Med* 2014; 161:319–327. 25047577 10.7326/M14-1027 PMC4236912

[2] Saez-Cirion A Bacchus C Hocqueloux L Avettand-Fenoel V Girault I Lecuroux C **Posttreatment HIV-1 controllers with a long-term virological remission after the interruption of early initiated antiretroviral therapy ANRS VISCONTI Study** . *PLoS Pathog* 2013; 9:e1003211. 23516360 10.1371/journal.ppat.1003211 PMC3597518

[3] Frange P Faye A Avettand-Fenoel V Bellaton E Deschamps D Angin M **HIV-1 virological remission lasting more than 12 years after interruption of early antiretroviral therapy in a perinatally infected teenager enrolled in the French Pediatric Cohort ANRS EPF-CO10. A case report** . *Lancet HIV* 2015; [In press]. 10.1016/S2352-3018(15)00232-5 26762993

[4] Luzuriaga K Gay H Ziemniak C Sanborn KB Somasundaran M Rainwater-Lovett K **Viremic relapse after HIV-1 remission in a perinatally infected child** . *N Engl J Med* 2015; 372:786–788. 25693029 10.1056/NEJMc1413931 PMC4440331

[5] Pereyra F Addo MM Kaufmann DE Liu Y Miura T Rathod A **Genetic and immunologic heterogeneity among persons who control HIV infection in the absence of therapy** . *J Infect Dis* 2008; 197:563–571. 18275276 10.1086/526786

[6] Jessen H Allen TM Streeck H **How a single patient influenced HIV research: 15-year follow-up** . *N Engl J Med* 2014; 370:682–683. 24521131 10.1056/NEJMc1308413 PMC4264571

[7] Goulder PJ Walker BD **HIV and HLA class I: an evolving relationship** . *Immunity* 2012; 37:426–440. 22999948 10.1016/j.immuni.2012.09.005 PMC3966573

[8] Ndhlovu ZM Proudfoot J Cesa K Alvino DM McMullen A Vine S **Elite controllers with low to absent effector CD8+ T cell responses maintain highly functional, broadly directed central memory responses** . *J Virol* 2012; 86:6959–6969. 22514340 10.1128/JVI.00531-12 PMC3393560

[9] Ndhlovu ZM Stampouloglou E Cesa K Mavrothalassitis O Alvino DM Li JZ **The breadth of expandable memory CD8+ T cells inversely correlates with residual viral loads in HIV elite controllers** . *J Virol* 2015; 89:10735–10747. 26269189 10.1128/JVI.01527-15 PMC4621138

[10] Payne RP Kloverpris H Sacha JB Brumme Z Brumme C Buus S **Efficacious early antiviral activity of HIV Gag- and Pol-specific HLA-B 2705-restricted CD8+ T cells** . *J Virol* 2010; 84:10543–10557. 20686036 10.1128/JVI.00793-10 PMC2950555

[11] Chen H Ndhlovu ZM Liu D Porter LC Fang JW Darko S **TCR clonotypes modulate the protective effect of HLA class I molecules in HIV-1 infection** . *Nat Immunol* 2012; 13:691–700. 22683743 10.1038/ni.2342 PMC3538851

[12] Lecuroux C Saez-Cirion A Girault I Versmisse P Boufassa F Avettand-Fenoel V **Both HLA-B∗57 and plasma HIV RNA levels contribute to the HIV-specific CD8+ T cell response in HIV controllers** . *J Virol* 2014; 88:176–187. 24131719 10.1128/JVI.02098-13 PMC3911721

[13] Frahm N Kiepiela P Adams S Linde CH Hewitt HS Sango K **Control of human immunodeficiency virus replication by cytotoxic T lymphocytes targeting subdominant epitopes** . *Nat Immunol* 2006; 7:173–178. 16369537 10.1038/ni1281

[14] Kiepiela P Ngumbela K Thobakgale C Ramduth D Honeyborne I Moodley E **CD8+ T-cell responses to different HIV proteins have discordant associations with viral load** . *Nature Med* 2007; 13:46–53. 17173051 10.1038/nm1520

[15] Janes H Friedrich DP Krambrink A Smith RJ Kallas EG Horton H **Vaccine-induced Gag-specific T cells are associated with reduced viremia after HIV-1 infection** . *J Infect Dis* 2013; 208:1231–1239. 23878319 10.1093/infdis/jit322 PMC3778967

